# Prophylactic potential of a *Panchgavya *formulation against certain pathogenic bacteria

**DOI:** 10.12688/f1000research.16485.1

**Published:** 2018-10-08

**Authors:** Pooja Patel, Chinmayi Joshi, Snehal Funde, Hanumanthrao Palep, Vijay Kothari

**Affiliations:** 1 Institute of Science, Nirma University, Ahmedabad, India; 2 Dr. Palep’s Medical Research Foundation, Mumbai, India

**Keywords:** Panchgavya, Prophylactic, Anti-infective, Caenorhabditis elegans

## Abstract

A *Panchgavya *preparation was evaluated for its prophylactic efficacy against bacterial infection, employing the nematode worm *Caenorhabditis elegans* as a model host. Worms fed with the *Panchgavya *preparation prior to being challenged with pathogenic bacteria had a better survival rate against four out of five test bacterial pathogens, as compared to the control worms. *Panchgavya *feeding prior to bacterial challenge was found to be most effective against *Staphylococcus aureus*, resulting in 27% (p=0.0001) better worm survival. To the best of our awareness, this is the first report demonstrating *in vivo* prophylactic efficacy of *Panchgavya* mixture against pathogenic bacteria.

## Introduction

*‘Panchgavya’* is a term used to describe a combination of five major substances obtained from cow, including cow’s urine, milk, ghee (clarified butter), curd and dung. Dhanvantari, referred to as the God of Indian Medicine, is said to have offered to mankind this wonder medicine called *Panchgavya*. In Sanskrit, all its five ingredients are individually called ‘Gavya’ and collectively termed as *Panchgavya* ( *panch* means five). *Panchgavya* products have been claimed to be beneficial in curing several human ailments, enhancing immunity and providing resistance to fight infections ( Dhama *et al.,* 2005). *Panchgavya* therapy (cowpathy) has been indicated as an alternate prophylactic and therapeutic modality for sound livestock and poultry health along with human health ( Dhama *et al*., 2014). *Panchgavya Prashan* is a common tradition followed by certain communities (e.g. Telugu Brahmins) in India, wherein a *Panchgavya* dose is taken once every year during monsoon season. The potential applications of *Panchgavya* as antimicrobials, immune boosters, antidiabetics, anticancer, anticonvulsant, aphrodisiac, blood purifiers, and as a suitable medium to deliver medicines, have caught the attention of scientists and medical professionals ( Dhama *et al*., 2014). In this context, we undertook an investigation on the prophylactic potential of a *Panchgavya* preparation against bacterial infections in the nematode host *Caenorhabditis elegans*.

## Methods

### *Panchgavya* preparation

The *Panchgavya* formulation used in this study was prepared using a method that was different from the one practiced traditionally (which yields a fermented preparation). Fresh cow dung and urine, sourced from a cow fed on cottonseed and sugarcane grass, were mixed thoroughly in a glass beaker. This mix was allowed to stand for 10 min and subjected to filtration through a muslin cloth (the traditional method does not involve filtration). To this filtrate, fresh cow’s milk and fresh curd was added, and mixed until a uniform mixture was formed. Finally, cow ghee was added to this mixture and mixed thoroughly.

Dung, urine, and milk were all sourced from a single cow. From the same batch of milk, curd and ghee were prepared. Cream of this milk was boiled for 30–40 min and filtered; the filtrate was taken as ghee. For curd preparation, one part of this milk was inoculated with previous batch of curd (prepared using milk from the same cow by adding few drops of lemon juice to the milk) followed by overnight incubation at room temperature.

The ratio of dung:urine:milk:curd:ghee in this preparation was 1:2:3:3:1. This *Panchagavya* mixture was then transferred to a copper vessel (covered with a muslin cloth) and allowed to rest for 30 min. This was followed by freeze-drying at -20 °C to convert the preparation in powder form, which was stored under refrigeration (4–8°C) until used for the microbiological experiments. When required for use, the *Panchgavya* powder was suspended in sterile distilled water to attain OD _625_ = 0.10±0.01.

### Test bacteria

Pathogenic bacteria used in this study included: *Staphylococcus aureus* (MTCC 737); beta-lactamase producing multidrug resistant strains of *Chromobacterium violaceum* (MTCC 2656) and *Serratia marcescens* (MTCC 97); multidrug resistant *Pseudomonas aeruginosa;* and *Streptococcus pyogenes* (MTCC 1924). *P. aeruginosa* was sourced from our internal culture collection *.* All other cultures were procured from MTCC (Microbial Type Culture Collection, Chandigarh, India).

### *In vivo* assay

*C. elegans* worms (received gift from the Biology Division, Sophia College, Mumbai) maintained on NGM (Nematode Growing Medium; 3 g/L NaCl, 2.5 g/L peptone, 1 M CaCl _2_, 1 M MgSO _4_, 5 mg/mL cholesterol, 1 M phosphate buffer of pH 6, 17 g/L agar-agar; this medium was prepared by us using the listed ingredients purchased from Merck, Mumbai or HiMedia, Mumbai) agar plate with *E. coli* OP50 (LabTIE B.V., JR Rosmalen, the Netherlands) as food, were kept unfed 24h prior to being used for experiments.

These worms were fed with *Panchgavya* by mixing this formulation (100 µL) with M9 medium (800 µL) and placed in a 24-well plate (sterile, non-treated polystyrene plates; HiMediaTPG24) containing 10 worms per well. Duration of exposure of worms to *Panchgavya* was kept 24, 48, 72 or 96 h, followed by addition of pathogenic bacteria (100 µL of bacterial suspension with OD _764_= 1.50). Appropriate controls i.e. worms previously not exposed to *Panchgavya*, but exposed to pathogenic bacteria; worms exposed neither to *Panchgavya* nor bacteria; and worms exposed to *Panchgavya*, but not to bacterial pathogens, were also included in the experiment. Incubation was carried out at 22°C.

Number of live vs. dead worms were counted every day for 5 days by putting the plate (with lid) under a light microscope (4X). Straight worms were considered to be dead. Plates were gently tapped to confirm lack of movement in the dead-looking worms. On the last day of the experiment, when plates could be opened, their death was also confirmed by touching them with a straight wire, wherein no movement was taken as confirmation of death.

### Statistical analysis

Values reported are means of four independent experiments, whose statistical significance was assessed using *t*-test performed through Microsoft Excel (2013). *P* values ≤0.05 were considered to be statistically significant.

## Results

**Figure 1.**
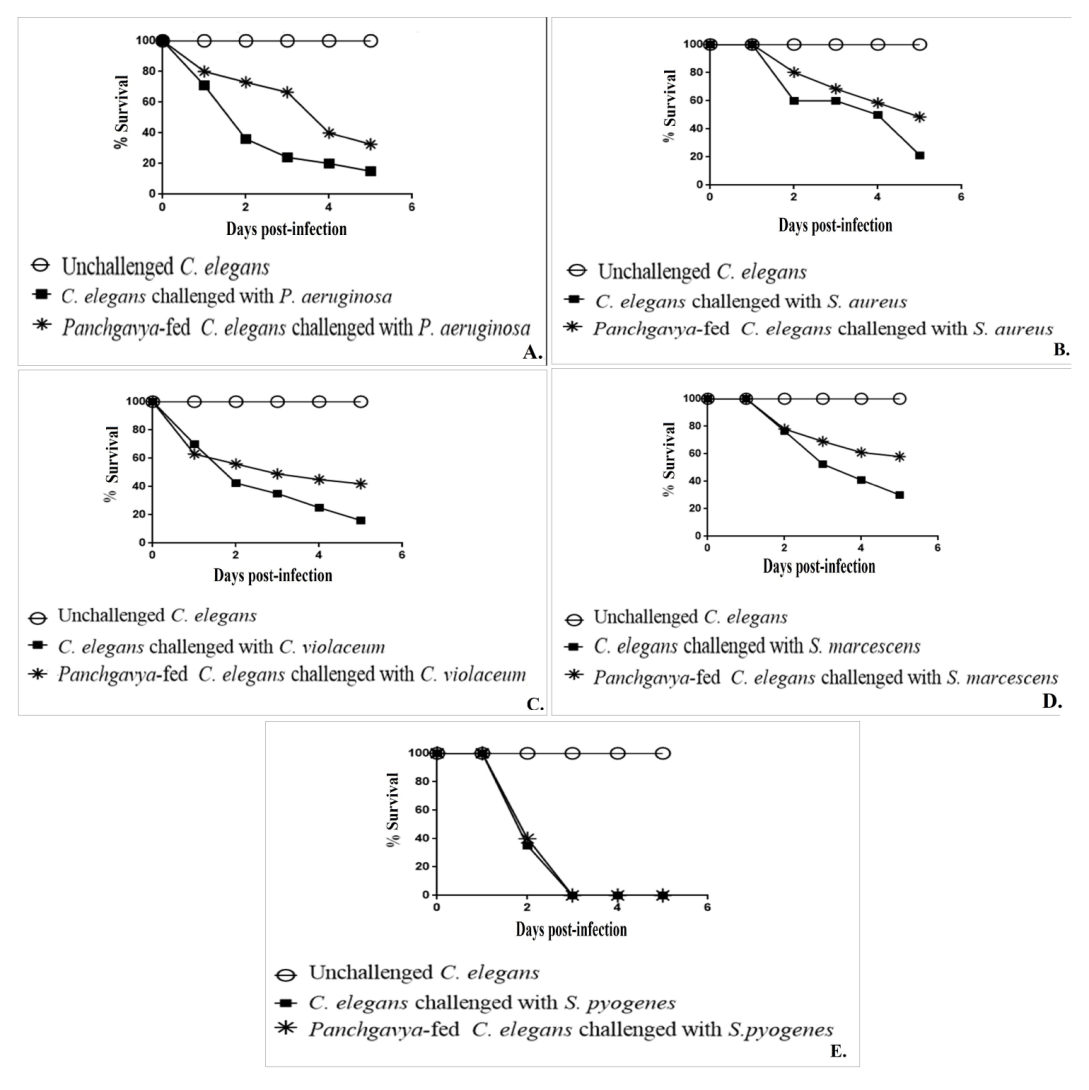
*Panchgavya*-exposed *Caenorhabditis elegans* exhibit better resistance to pathogenic bacteria. Previous exposure to *Panchgavya* (for 72 or 96 h) enabled *C. elegans* population to register better survival in the face of bacterial challenge: ( **A**) 42.50±2.52% (p=0.001) better survival till third day, and 17.50±3.54% (p=0.002) better survival on fifth day, against *P. aeruginosa*; ( **B**) 27.30±1.86% (p=0.0001) better survival on fifth day, against *S. aureus*; ( **C**) 21.50±1.04% (p=0.0003) better survival on fifth day, against *C. violaceum*; ( **D**) 23±1.50% (p=0.002) higher survival on fifth day, against *S. marcescens*; ( **E**) *Panchgavya*-exposure was not found to confer any protection on *C. elegans* against *S. pyogenes* challenge. Results pertaining to 72 h and 96 h exposure of worms to *Panchgavya,* prior to bacterial challenge, were not statistically different. Values reported are means of four independent experiments, whose statistical significance was assessed using *t*-test performed through Microsoft Excel. *P* values ≤0.05 were considered to be statistically significant.

Worms fed on *Panchgavya* for 24 or 48 h registered no different (p>0.05) survival rates in the face of bacterial challenge as compared to control worms ( Appendix A and Appendix B). However, worms with 72 or 96 h *Panchgavya* exposure registered a 15–27% (p<0.05) better survival upon challenge with different pathogenic bacteria, except for *S. pyogenes* as compared with control worms ( Figure 1; Appendix C and Appendix D). These results demonstrate the prophylactic potential of *Panchgavya* against four different gram-positive and gram-negative bacterial infections, wherein previous exposure of *C. elegans* to this formulation was found to confer statistically significant protection on this worm against subsequent bacterial attack.

However, when administered to *C. elegans* already infected by these pathogens, *Panchgavya* was not found to offer any survival benefit to the nematode host ( Appendix E). Additionally, the *Panchgavya*-exposed worm population was able to generate progenies in absence as well as presence of pathogenic bacteria, which did not happen in control wells containing *Panchgavya*-unexposed worms, suggesting overall higher fitness of *Panchgavya*-exposed worms.

Raw data has been provided in Appendices A-E Appendix A: Bacterial challenge to *C. elegans* fed on *Panchgavya* for 24 h; Appendix B: Bacterial challenge to *C. elegans* fed on *Panchgavya* for 48 h; Appendix C: Bacterial challenge to *C. elegans* fed on *Panchgavya* for 72 h; Appendix D: Bacterial challenge to *C. elegans* fed on *Panchgavya* for 96 h; Appendix E: *Panchgavya* tested as a therapy for already infected *C. elegans*
Copyright: © 2018 Patel P et al. 2018 Data associated with the article are available under the terms of the Creative Commons Zero "No rights reserved" data waiver (CC0 1.0 Public domain dedication).

## Conclusions

Though there are few reports mentioning *in vitro* antimicrobial activity of either *Panchgavya* mixture ( Gajbhiye *et al.*, 2018) or its individual components ( Deepika *et al*., 2016), to the best of our knowledge, the present study is the first report demonstrating *in vivo* anti-infective efficacy of *Panchgavya* mixture. The observed protective effect of *Panchgavya* against bacterial infection may in part stem from its immunomodulatory potential ( Gajbhiye *et al*., 2015). This short study validates the therapeutic potential of *Panchgavya* mentioned in *Ayurved* ( Susruta Samhita, 1885). Further studies for characterization (e.g. generating its metagenomic, which may reveal presence of beneficial microbes, and chemical profile) of this ancient formulation can provide insights into the mechanisms underlying its anti-infective efficacy.

## Data availability

The data referenced by this article are under copyright with the following copyright statement: Copyright: © 2018 Patel P et al.

Data associated with the article are available under the terms of the Creative Commons Zero "No rights reserved" data waiver (CC0 1.0 Public domain dedication). 

F1000Research: Dataset 1. Raw data has been provided in Appendices A-E., http://dx.doi.org/10.5256/f1000research.16485.d220622 ( Patel *et al*., 2018).

- Appendix A: Bacterial challenge to *C. elegans* fed on *Panchgavya* for 24 h
- Appendix B: Bacterial challenge to *C. elegans* fed on *Panchgavya* for 48 h
- Appendix C: Bacterial challenge to *C. elegans* fed on *Panchgavya* for 72 h
- Appendix D: Bacterial challenge to *C. elegans* fed on *Panchgavya* for 96 h
- Appendix E: *Panchgavya* tested as a therapy for already infected *C. elegans*
