# Supplementary material for: Prophylactic potential of a Panchgavya formulation against certain pathogenic bacteria
Source: F1000Res. 2018 Oct 8;7:1612. [Version 1] doi: 10.12688/f1000research.16485.1 (PMC6206607; doi:10.12688/f1000research.16485.1)
Supplement: Peer review history

# Peer review: Referee response for version 1

**DOI:** 10.5256/f1000research.18019.r39648

**Published:** 2018-12-31

**Authors:** Neha Jain

**Affiliations:** 1 Biological and Life Sciences, School of Arts and Sciences, Ahmedabad University, Ahmedabad, Gujarat, India

The study on *Panchgavya* by Patel *et al*. is an interesting observation. The formulation seems to have positive effect on the worms with bacterial infections, however, at this point the authors have not elucidated the mechanism. A follow-up study could answer the following questions:

 1. What are the genes that *Panchgavya* may target?

 2. Is the protection by killing the bacteria?

 3. Does *Panchgavya* change antibiotic resistant patterns in the bugs used in the study?

 4. Can it be used with polymicrobial infections and biofilm forming bacteria?

 Additional comments:

*1. The study design is appropriate and the work is technically sound.*

*2. The conclusions drawn are adequately supported by the results.*

I have read this submission. I believe that I have an appropriate level of expertise to confirm that it is of an acceptable scientific standard.

# Peer review: Referee response for version 1

**DOI:** 10.5256/f1000research.18019.r39214

**Published:** 2018-10-26

**Authors:** Prasun Kumar

**Affiliations:** 1 Department of Chemical Engineering, Chungbuk National University, Cheongju, South Korea

Antibiotic-resistant infections are a global threat, and novel antibiotic, as well as non-antibiotic approaches to deal with the problem of antimicrobial resistance (AMR), are urgently needed. In this pursuit, taking insights from traditional wisdom seems to be a logical effort. This particular research note describes an investigation of the prophylactic efficacy of a traditional Indian formulation - *Panchgavya*, and using the nematode worm model has demonstrated such a prophylactic effect to be there against some of the pathogenic bacteria. I appreciate that they have selected important pathogens like *P. aeruginosa*, and *S. marcescens *(Enterobacteriaceae). 

 Despite *Panchgavya *being an ancient formulation, it seems to be under-investigated by modern-day scientists. Studies like this one attempting to validate the traditional medicine claims are welcome. As mentioned in the conclusion part by authors, they should take up in near future further characterization of this formulation. If such a standardized formulation can be made available for public use, it may help in reducing the overall infection burden of human populations.

 - The formulation concentration [OD _625_ = 0.10±0.01], is it right? The authors may use w/v units for clarity.

 - Will there be any effect of different doses of *Panchgavya?*

 - There are many interesting questions that are yet to be addressed, most possibly by taking up this research further and analyzing the mechanism, bioactive component etc. 

 - An independent experiment similar to MIC using a CFU-based method for each of the selected pathogens would have shed more light. 

 - Differential behavior on Gram +ve bacteria is quite interesting and must be studied further.

I have read this submission. I believe that I have an appropriate level of expertise to confirm that it is of an acceptable scientific standard.

# Peer review: Referee response for version 1

**DOI:** 10.5256/f1000research.18019.r39211

**Published:** 2018-10-19

**Authors:** Subramani Parasuraman

**Affiliations:** 1 Department of Pharmacology, Faculty of Pharmacy, AIMST University, Bedong, Malaysia

- Authors have used beta-lactamase producing antibiotic-resistant strains in their study. That is good, as resistant strains of *P. aeruginosa* and *S. marcescens* have also been listed by WHO as of high priority.
- Use of *C. elegans* as a model host by authors is also logical, as there is some overlap among the virulence factors of pathogenic bacteria damaging *C. elegans*, and those damaging human cells.
- While the current study may be approved for publication as a research note, in future authors should try to investigate the mechanisms through which *'Panchgavya*' imparts protection to *C. elegans* against infectious bacteria.

I have read this submission. I believe that I have an appropriate level of expertise to confirm that it is of an acceptable scientific standard.
